# Dosimetric evaluation of patient-side range shifters using two materials for superficial region treatment in spot scanning proton therapy: impact of material, air gap and setup distance

**DOI:** 10.1093/jrr/rrag058

**Published:** 2026-07-23

**Authors:** Seiichi Ota, Tomohiro Kajikawa, Toshiyuki Ogata, Hisashi Takaya, Kenta Kijima, Tetsuya Okita, Norihiro Aibe

**Affiliations:** Division of Radiological Technology, Department of Medical Technology, University Hospital, Kyoto Prefectural University of Medicine, 465 Kajii-cho, Kawaramachi-Hirokoji, Kamigyo-ku, Kyoto 602-8566, Japan; Department of Radiology, Kyoto Prefectural University of Medicine, 465 Kajii-cho, Kawaramachi-Hirokoji, Kamigyo-ku, Kyoto 602-8566, Japan; Department of Radiology, Kyoto Prefectural University of Medicine, 465 Kajii-cho, Kawaramachi-Hirokoji, Kamigyo-ku, Kyoto 602-8566, Japan; Division of Radiological Technology, Department of Medical Technology, University Hospital, Kyoto Prefectural University of Medicine, 465 Kajii-cho, Kawaramachi-Hirokoji, Kamigyo-ku, Kyoto 602-8566, Japan; Division of Radiological Technology, Department of Medical Technology, University Hospital, Kyoto Prefectural University of Medicine, 465 Kajii-cho, Kawaramachi-Hirokoji, Kamigyo-ku, Kyoto 602-8566, Japan; Division of Radiological Technology, Department of Medical Technology, University Hospital, Kyoto Prefectural University of Medicine, 465 Kajii-cho, Kawaramachi-Hirokoji, Kamigyo-ku, Kyoto 602-8566, Japan; Department of Radiology, Kyoto Prefectural University of Medicine, 465 Kajii-cho, Kawaramachi-Hirokoji, Kamigyo-ku, Kyoto 602-8566, Japan

**Keywords:** range shifter, air gap, proton therapy, spot scanning, superficial target, immobilization

## Abstract

This study aimed to evaluate the dosimetric characteristics of patient-side range shifters (RS) made of polymethyl methacrylate and high-density polyethylene for superficial region treatment in spot scanning proton therapy. The impact of air gap (AG) size and RS setup deviations on dose accuracy was investigated. A virtual water phantom and a simple volumetric beam arrangement were used for dose calculations in a treatment planning system employing a pencil beam algorithm with a triple Gaussian kernel. Gamma passing rates (GPRs) and point dose measurements were performed using a two-dimensional detector array and an ionization chamber, with AGs ranging from 0 to 110 mm. Results showed that GPRs exceeded 90% at depths of 9, 20 and 50 mm when the AG was within 80 mm, even under 2%/2 mm evaluation criteria. At AGs of 90–110 mm, reduced GPRs and >3% deviations in point dose measurements were observed at shallow depths. Setup deviation experiments were conducted by intentionally shifting the RS slab from the geometry used for reference measurements, and GPRs were evaluated relative to the corresponding measured reference at each original AG. The results indicated that shifts of up to ±10 mm from the reference AG had minimal dosimetric impact, with GPRs remaining above 96.8% under 1%/1 mm criteria. Maintaining the AG within 80 mm is recommended to ensure dosimetric accuracy. These findings suggest that patient-side RS systems can be a practical option in clinical scenarios where nozzle-mounted RS devices are not feasible.

## INTRODUCTION

In the spot scanning technique, monoenergetic proton beams are directed to specific points within the target, and the full dose distribution is achieved by combining multiple beam energies. However, commercial proton therapy systems typically have a minimum deliverable proton energy of ~70 MeV [1], which limits the ability to treat superficial tumors directly. To overcome this limitation, range shifters (RS) are commonly used to pull back the proton range for shallow targets. Conventional nozzle-mounted RS devices, which are attached to the treatment nozzle, are widely employed in modern proton therapy systems [2, 3]. These systems are typically modeled in the treatment planning system (TPS) and are used in most clinical settings.

However, the use of nozzle-mounted RS devices can be challenging in specific clinical scenarios, particularly when a dorsal (posterior) beam arrangement is required. In such cases, there is a potential risk of collision with the treatment couch, limiting the flexibility of beam angles. To address these constraints, patient-side RS systems—such as couch-top boluses and third-party RS devices—have been introduced as practical alternatives that can be positioned closer to the patient without interfering with the treatment equipment [4, 5]. Unlike nozzle-mounted RS, these patient-side RS devices are not always supported in commercial TPS, which may pose challenges in dose calculation accuracy and quality assurance.

This study investigates patient-side RS, specifically two types made of polymethyl methacrylate (PMMA) and high-density polyethylene (HDPE), which were selected for their widespread availability and stable physical properties. These materials were also chosen based on their scattering characteristics: PMMA exhibits scattering properties similar to those of water, while HDPE shows less scattering compared to water [6]. From an image-guidance perspective, we also prioritized low-density materials (PMMA: 1.19 g/cm^3^; HDPE: 0.97 g/cm^3^), since a higher-density couch-top bolus material (G-10 fiberglass; 1.85 g/cm^3^) has been reported to increase cone-beam CT (CBCT) artifacts [4]. Slab thickness was also considered relevant from a dose-calculation perspective, because a previous study suggested that thicker range shifters may lead to larger dosimetric deviations in pencil beam treatment planning [7]. Accordingly, we evaluated clinically feasible slab thickness options (PMMA: 31/36 mm; HDPE: 36/42 mm) representing practical designs with different water-equivalent thicknesses.

These materials were commissioned based on monoenergetic proton beam measurements in shallow regions following the method of Yasui et al. [2]. The percentage differences in beam spot sizes in water for the two materials are provided in Supplementary Fig. S1.

The primary objective of this study was to evaluate the dosimetric effects of using patient-side RS, particularly the impact of air gap (AG) size between the RS and the patient surface, as well as setup position deviations. A simple beam arrangement was used to simulate the irradiation of a volumetric (three-dimensional) target. Through this patient-side RS approach, we aim to provide practical data to support its clinical application in situations where nozzle-mounted RS devices cannot be used, without performing direct comparisons to conventional nozzle-mounted systems. We hypothesized that the dosimetric accuracy of patient-side RS may degrade beyond a specific AG threshold.

## MATERIALS AND METHODS

### Geometry and beam setting for treatment planning calculations

A single beam that covered a simple volumetric target was perpendicularly directed into a virtual water phantom in use of VQA [1, 2, 8, 9] ver. 5.6.1 (Hitachi Ltd., Japan) as a TPS, that commissioned for PROBEAT-CR (Hitachi Ltd., Japan) as spot scanning proton therapy unit. Isocenter was positioned 50 mm below the surface of the virtual water phantom, and a target volume was defined as 80 × 80 mm laterally and 80 mm in depth, extending from the water surface to the distal edge of the target (Fig. 1a). The AG between a RS slab and the water phantom surface were set from 0 to 110 mm in 10 mm increments. A prescription dose of 1.1 Gy (RBE), corresponding to a physical dose of 1.0 Gy, was assigned to the target. Dose calculation was performed using single-field uniform dose optimization with a calculation grid size of 1 × 1 × 1 mm and a spot spacing interval of 5 mm. A total of 53 beam energies ranging from 72.1 to 130.2 MeV, corresponding to nominal ranges of 41.6 to 122.1 mm, were used. A pencil beam algorithm with a triple Gaussian kernel model [10] was employed for dose calculation. Subsequently, for each AG, dose recalculations were performed using the same beam settings.

**Fig. 1:**
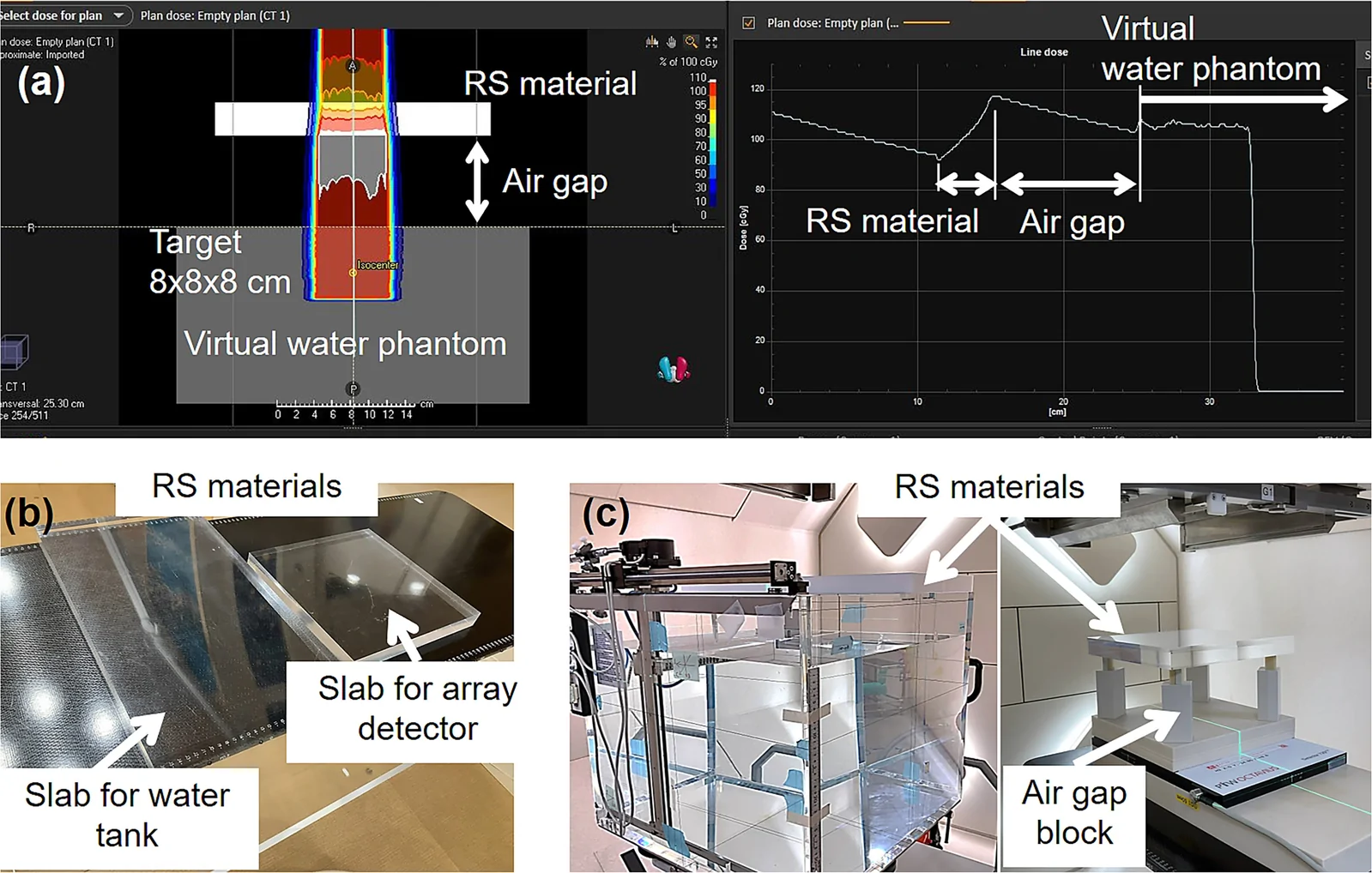
(a) Beam setting in treatment planning (VQA, Hitachi Ltd., Japan); (b) range shifter slabs for water tank measurement and array detector measurement; (c) measurement setup including range shifter slabs.

The physical densities of the RS materials used in this study were 1.19 g/cm^3^ for PMMA (Sumitomo Chemical Co., Ltd., Japan) and 0.97 g/cm^3^ for HDPE (Takiron Polymer Co., Ltd., Japan). RS slabs with physical thicknesses of 31 and 36 mm for PMMA, and 36 and 42 mm for HDPE, were used. The water equivalent ratios (WERs) [11] of PMMA and HDPE were 1.162 ± 0.005 and 1.036 ± 0.005 (Mean ± SD), respectively. These values were determined by acquiring integrated depth dose curves with and without the RS slab in advance and calculating the ratio of the difference in range (defined as distal 90%) to the physical thickness of the RS slab. Each measurement was performed at least 10 times. The mean WERs were used in treatment planning.

### Data acquisition for comparison with TPS calculations

For TPS-to-measurement comparisons, measurements were performed using the same AG values as those set in the TPS for each RS condition.

### Two-dimensional dose measurements

For comparison between TPS calculations and measurements, an Octavius 729 (PTW, Germany), a 2D detector array with a 9 mm water-equivalent entrance thickness, was used. Gamma passing rate (GPR), based on global gamma analysis using 2%/2 mm criteria and a 10% dose threshold, was evaluated at three measurement depths: 9, 20 and 50 mm. A tough water phantom (Kyoto Kagaku, Japan) with a lateral size of 300 × 300 mm was used as the water-equivalent phantom to adjust the measurement depth. To ensure reproducibility of the AG, supporting blocks were used to hold the RS slab in place (Fig. 1b and c). At least three measurements were performed for each combination of conditions.

### Point dose measurements

Point dose measurements along the central beam axis in water were performed using an MP-3 water tank (PTW, Germany), an Advanced Markus ionization chamber (PTW, Germany) and a Unidos electrometer (PTW, Germany). For these measurements, the RS slab with a lateral size of 300 × 650 mm was used so that it could be placed on top of the water tank (Fig. 1b and c). The AG between the RS and the water surface was carefully measured and set using a ruler. Measurement depths in water were set at 3, 5, 7, 9, 10, 20, 35, 50 and 70 mm, as well as at a minimum of four points in distal fall-off region along the central axis. These measurements were also used for comparison with TPS-calculated doses.

### Impact of RS setup deviations: measurement-to-measurement comparison

To evaluate the dosimetric impact of RS slab setup deviations, measurements were performed using the 2D detector array with intentional AG offsets in 10 mm increments from 0 to 110 mm. The original AGs were set to 30, 50 and 70 mm. Instead of the TPS-calculated dose distributions, measurements acquired at the original AGs served as the reference for the global gamma analysis (1%/1 mm). To minimize daily output variations, all measurements were performed consecutively on the same day. The RS materials were PMMA (36 mm) and HDPE (42 mm), and measurements were performed at depths of 9, 20 and 50 mm.

### Statistical analysis

The GPRs from the 2D detector array were compared using the Kruskal–Wallis test with Dunn’s test for post hoc analysis. A *P*-value <0.05 was considered significant. Analyses were performed in GraphPad Prism v9.5.1 (GraphPad Software, USA).

## RESULTS

In point dose measurements, differences exceeding 3% between the measurements and the TPS were observed at shallow depths (e.g. 3 and 5 mm) when the AG was set to 100 mm (Fig. 2a–d). In the measurement of the distal fall-off region, large differences were observed, ranging from −25.5 to 166.7% in terms of local dose comparison; the maximum absolute dose difference was 0.27 Gy. However, the corresponding distance-to-agreement values were within 1 mm for each combination of RS and AG. At the overlapping depths of 9, 20 and 50 mm, the differences between the point dose measurements in water and the central-axis detector channel of the 2D detector array (water-equivalent phantom) across the evaluated AG settings were 0.6 ± 0.7%, 0.4 ± 0.8% and 0.0 ± 0.6%, respectively (data not shown).

**Fig. 2:**
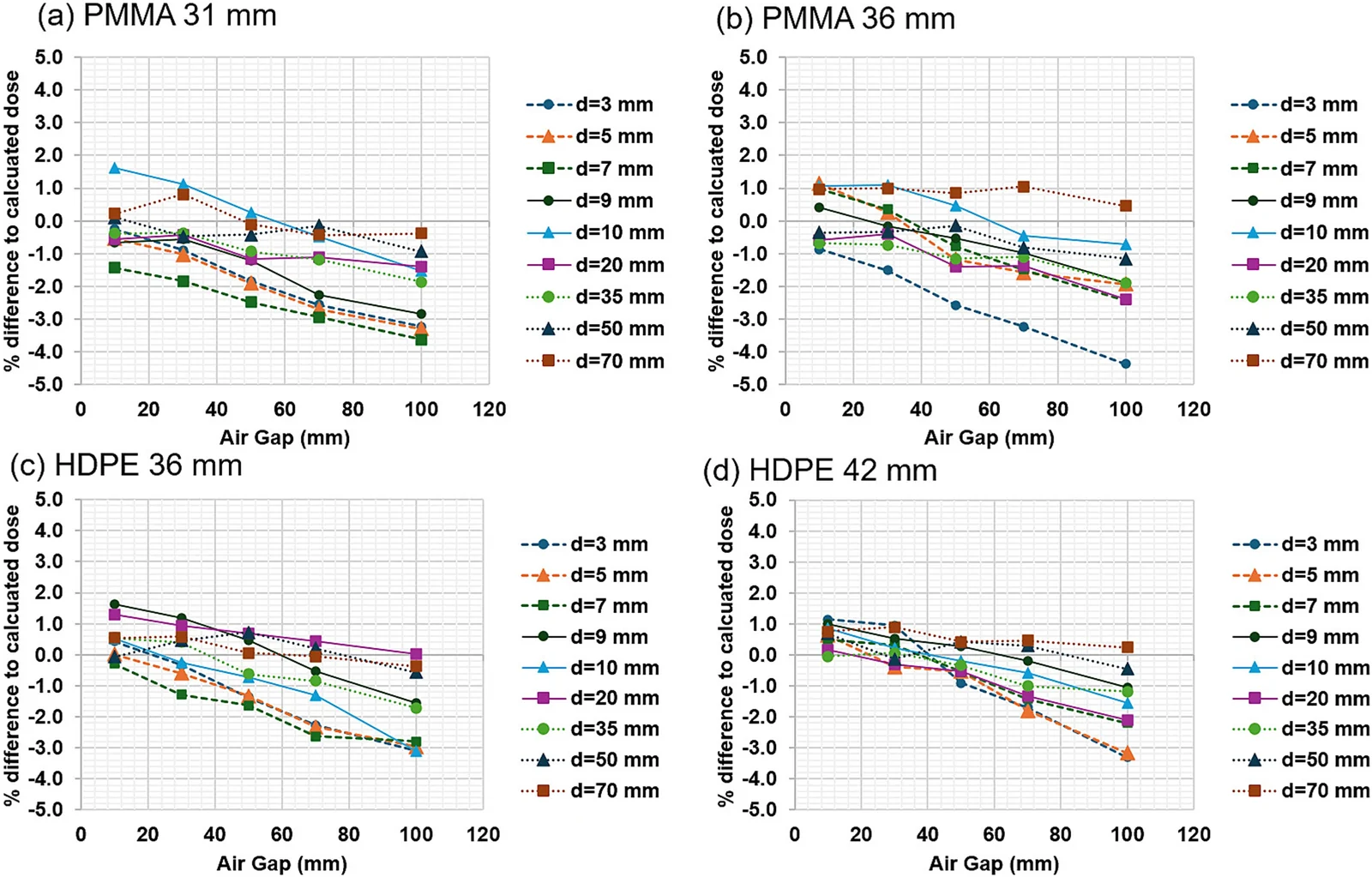
Percentage point dose differences between treatment planning and measured dose in water, using (a) PMMA 31 mm, (b) PMMA 36 mm, (c) HDPE 36 mm and (d) HDPE 42 mm as range shifter slabs.

In the gamma evaluation, AG values were grouped into four ranges (0–20, 30–50 mm, 60–80 mm and 90–110 mm), with each group comprising at least nine measurements. In the AG groups of 0–20 mm, 30–50 mm and 60–80 mm, the mean GPRs (2%/2 mm criteria) at depths of 9, 20 and 50 mm all exceeded 90%. Although statistically significant differences were observed between these and the other groups (Fig. 3a–l), the GPRs remained above 90% across these measurement conditions. In thicker RS slabs of PMMA (36 mm) and HDPE (42 mm), the mean GPRs fell below 90% under the combination of AG group (90–110 mm) and shallow depths (9 and 20 mm) (Fig. 3d, e, j and k). These groups also showed a tendency toward larger standard deviations. Statistically significant differences were observed between these and other groups (Fig. 3d, e and k).

**Fig. 3:**
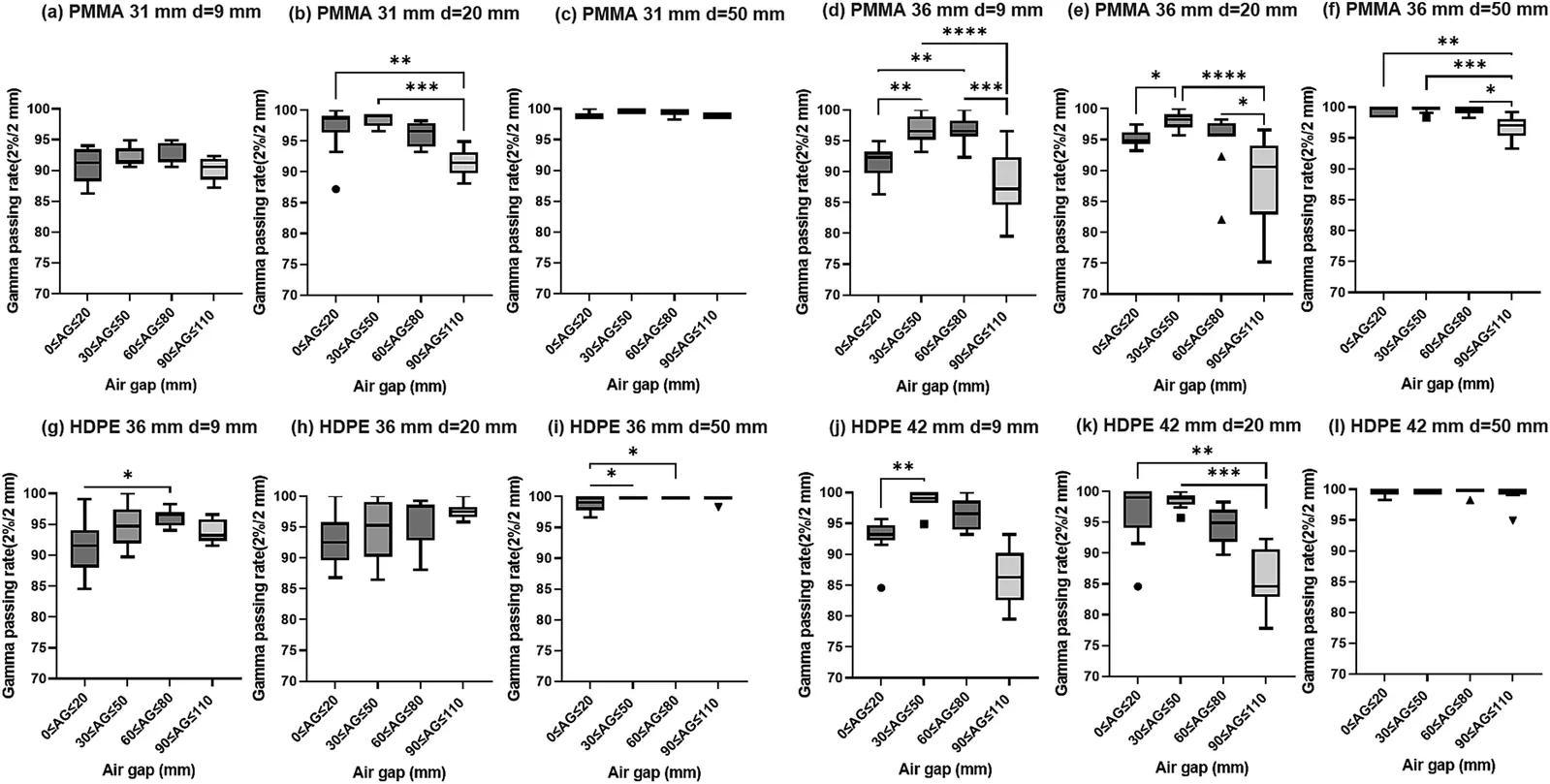
Gamma passing rate (2%/2 mm) as a function of range shifter material combination, thickness, AG and measurement depth. Panels (a–c), (d–f), (g–i), and (j–l) show results for PMMA 31-mm slabs, PMMA 36-mm slabs, HDPE 36-mm slabs, and HDPE 42-mm slabs, respectively. Within each material and thickness condition, the panels correspond to measurement depths of 9, 20, and 50 mm. Box-and-whisker plots follow the Tukey method, with whiskers representing 1.5 times the interquartile range. Significance levels: ^*^, *P* < 0.05; ^**^, *P* < 0.01; ^***^, *P* < 0.001 and ^****^, *P* < 0.0001.

As part of the setup shift evaluation using RS slabs made of PMMA (36 mm) and HDPE (42 mm), GPR was assessed with original AG values of 30, 50 and 70 mm. Setup shifts were applied to each AG. At a depth of 9 mm, AG shifts had a greater impact; however, GPR under the 1%/1 mm criteria remained above 96.8% at all depths within ±10 mm of the original AG. (Fig. 4a and b).

**Fig. 4:**
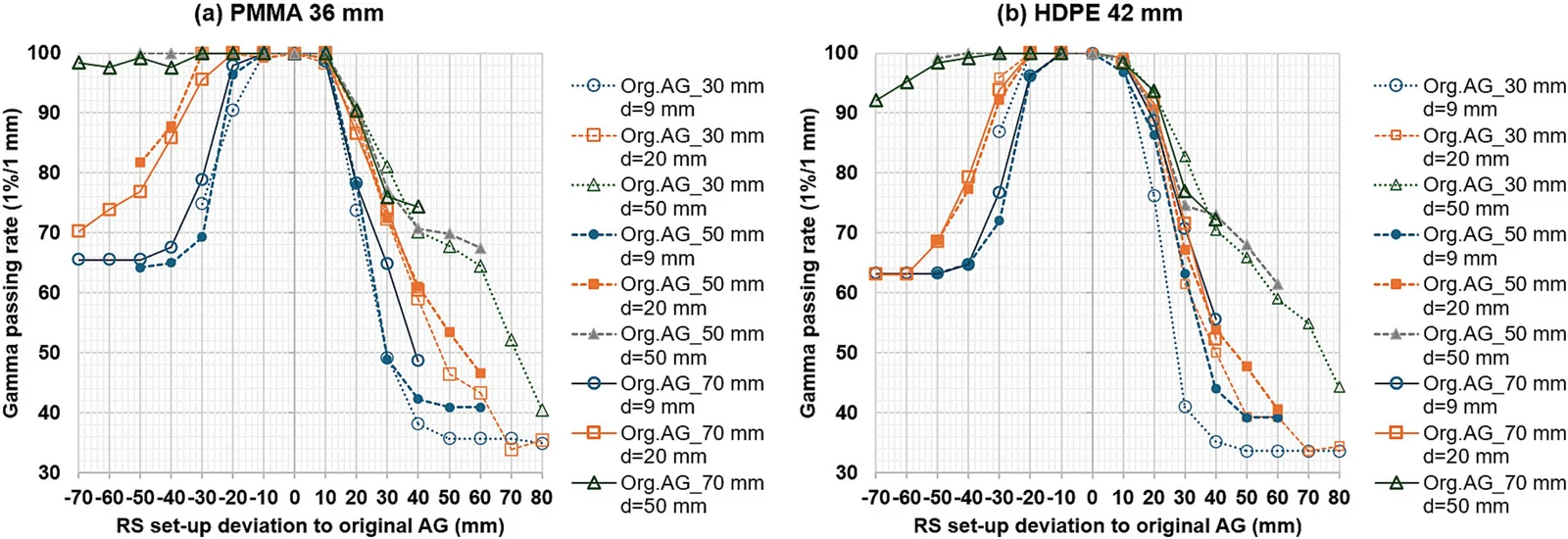
Evaluation of setup shifts in the RS slab using (a) PMMA (36 mm) and (b) HDPE (42 mm). Gamma passing rate (1%/1 mm) assessments were performed using the original AG as the reference, with setup shifts applied to each AG setting. Negative values on the horizontal axis indicate shifts toward the surface (i.e. closer to the detector). Org., original.

## DISCUSSION

While some statistically significant differences were observed between groups (Fig. 3a–l), the mean GPRs at depths of 9, 20 and 50 mm remained above 90% for AG values of 0–80 mm when using PMMA (36 mm) and HDPE (42 mm), and were thus considered acceptable. With thinner RS slabs (PMMA 31 mm and HDPE 36 mm), GPRs exceeded 90% across all combinations of AG and depth. Although the 2%/2 mm, 90% passing criterion is considered strict [12], it was adopted as the acceptance threshold in this evaluation. In contrast, mean GPRs fell below 90% at shallow depths (9 and 20 mm) when the AG was 90–110 mm, with greater standard deviations than those observed with smaller AG settings. Conversely, at a depth of 50 mm, GPRs approached 100%. These results support maintaining the AG below 80 mm for superficial targets, consistent with a previous study [7]. From a practical perspective, careful attention during CT simulation and patient immobilization may help keep the AG between the patient surface and the RS within 80 mm. Point dose measurements also showed >3% deviations at shallow depths (e.g. 3 and 5 mm) with an AG of 100 mm (Fig. 2), supporting the recommendation to minimize AG.

Consistency between point dose and central-axis 2D-array measurements at depths of 9, 20 and 50 mm was supported by mean differences <1% (SD ≤0.8%). These SD values represent the repeat-measurement variability, and additional uncertainty sources include detector calibration, positioning and AG setting.

A measurement-related limitation of this study was the 10 mm detector spacing of the 2D detector array used for gamma evaluation, resulting in limited spatial resolution that may have limited the precise assessment of fine spatial dose features. Because the lateral dose distribution in spot scanning proton therapy is formed by the overlap of multiple spots, the 2D-array results should be interpreted mainly as reflecting the relative effects of AG and material conditions at the plan level, rather than as a precise evaluation of individual spot penumbra or small-field characteristics.

One report indicated that changes in spot size of less than 25% from the nominal value have only a moderate or negligible impact on dose coverage [13]. In monoenergetic beam measurements of this study, the percent difference in 1σ spot sizes in water between measured and TPS-calculated values tended to increase with larger AG. At the AG of 100 mm, the differences were ~15% for 116.4 and 130.2 MeV beams and ~25% for the lower energy 84.4 MeV (Supplementary Fig. S1). In the superficial region, where low-energy components contribute more significantly, the spot size difference is more likely to approach 25%. This may have led to the reduced GPRs observed at shallow depths such as 9 and 20 mm under AG of 90–110 mm, as shown in Fig. 3.

These observations underscore the potential influence of calculation model characteristics, particularly in relation to the nuclear halo effect at large AGs, on dose calculation discrepancies between TPS-calculated and measured doses [7]. Here, the nuclear halo effect denotes the broad low-dose tail around the primary proton beam caused by nuclear interactions. The pencil beam algorithm with the triple Gaussian kernel model [10] used in this study is not widely implemented in commercially available TPSs, where single or double Gaussian models [7, 14] are more commonly adopted. The triple Gaussian model incorporates an additional low-dose component to better approximate the nuclear halo effect; however, a previous study [9] indicated that the differences between the triple and double Gaussian models are generally small, condition-dependent and particularly limited in shallow regions. This previous finding suggests that the discrepancies observed in this study may not be unique to the triple Gaussian model; however, further validation in other treatment planning systems is warranted. Although the observed discrepancies were interpreted mainly in relation to the nuclear halo effect, other mechanisms may also have contributed. In particular, changes in lateral scatter equilibrium at larger AGs, as well as material-dependent scattering behavior at interfaces [6], may have influenced the measured dose distribution. Because the present study was not designed to separately evaluate these factors, their respective contributions remain uncertain.

Monte Carlo simulations [7, 14] may reduce the AG-related discrepancies in shallow regions, but additional verification is required. The present findings may serve as a useful reference for clinical practice where pencil beam algorithms are employed.

In a separate measurement-to-measurement analysis assessing setup robustness, introducing a known AG offset of ~±10 mm from the original setup resulted in no substantial dosimetric change under the evaluated conditions (Fig. 4). This finding suggests that day-to-day variations in the RS-to-surface distance within ~±10 mm may be acceptable.

When the patient-side RS is placed laterally, as with a U-shaped bolus [5], or posteriorly, as with a couch-top [4], precise reproduction of the planned AG may be difficult, even with immobilization devices such as vacuum cushions and thermoplastic masks. However, our results indicate that a deviation of ~±10 mm from the planned AG can be tolerated without large dosimetric impact (Fig. 4). Nevertheless, careful management of the RS-to-patient distance, with in-room CBCT or verification CT scans [15] as needed, remains important in clinical practice.

In contrast, nozzle-mounted RS systems [2] may make position verification more difficult because such applicators typically need to be installed after imaging. Unless specifically designed with geometric considerations [3], these RS systems may interfere with the acquisition of position verification images during treatment, particularly when repeated position verification is required. This issue is especially relevant in pediatric cases, where treatment times are generally longer [16]. In this context, patient-side RS using low-density materials may offer practical workflow advantages, although their impact on image quality was not evaluated in this study.

However, these interpretations should be considered with several study limitations in mind. First, the present evaluation was performed using a specific pencil beam algorithm with a triple Gaussian kernel model, and the extent to which the findings can be generalized to other commercially available dose calculation algorithms, including double Gaussian and Monte Carlo-based models, remains uncertain. Second, the experimental conditions were intentionally simplified to isolate the effects of AG and to minimize confounding from other geometric or anatomical factors. While this design was advantageous for clarifying the specific influence of AG, it did not address more complex clinical situations, such as oblique beam incidence or anatomically heterogeneous media, and therefore limits the direct generalizability of the findings to actual patient treatments. Third, the potential effects of the evaluated materials on CBCT image quality were not assessed, nor were the influences of material aging, fabrication tolerances or long-term stability or temperature-related dimensional changes. These factors should be addressed in future studies to further clarify the robustness and clinical applicability of the present findings.

## CONCLUSIONS

The two types of materials for patient-side RS used in this study showed good agreement between treatment planning and measurements under the present conditions when the AG was within 80 mm. In daily treatment sessions, setup deviations of up to 10 mm from the planned AG between the beam entry surface and the RS may be acceptable.

## Supplementary Material

Supplementary_Fig_1_diff_of_1_sigma_in_water_Hi_res_rrag058

## Data Availability

The datasets generated and/or analyzed during the current study are available from the corresponding author on reasonable request.

## PRESENTATION AT A CONFERENCE

A part of this research was presented at the 23rd Asia-Australasia Conference of Radiological Technologists (2021) and Seoul Radiological Technologists Association International Conference (2022).
